# Enhancement of fermentable sugar yield by competitive adsorption of non-enzymatic substances from yeast and cellulase on lignin

**DOI:** 10.1186/1472-6750-14-21

**Published:** 2014-03-20

**Authors:** Yong Tang, Fuhou Lei, Carrasco Cristhian, Zuguang Liu, Hailong Yu, Jianxin Jiang

**Affiliations:** 1 Department of Chemistry and Chemical Engineering, Beijing Forestry University, Beijing, China; 2 GuangXi Key Laboratory of Chemistry and Engineering of Forest Products, Nanning 530006, China; 3 Department of Chemical Engineering, Lund University, Lund, Sweden

**Keywords:** Cellulases, Enzymatic hydrolysis, Yeast hydrolysate, Competitive adsorption

## Abstract

**Background:**

Enhancement of enzymatic digestibility by some supplementations could reduce enzyme loading and cost, which is still too high to realize economical production of lignocellulosic biofuels. A recent study indicates that yeast hydrolysates (YH) have improved the efficiency of cellulases on digestibility of furfural residues (FR). In the current work, the components of YH were separated by centrifugation and size exclusion chromatography and finally characterized in order to better understand this positive effect.

**Results:**

A 60.8% of nitrogen of yeast cells was remained in the slurry (YHS) after hydrothermal treatment. In the supernatant of YH (YHL), substances of high molecular weight were identified as proteins and other UV-absorbing compounds, which showed close molecular weight to components of cellulases. Those substances attributed to a synergetic positive effect on enzymatic hydrolysis of FR. The fraction of YHL ranged from 1.19 to 2.19 mL (elution volume) contained over 50% of proteins in YHL and had the best performance in stimulating the release of glucose. Experiment results proved the adsorption of proteins in YHL on lignin.

**Conclusions:**

Supplementation of cellulases with YH enhances enzymatic digestibility of FR mainly by a competitive adsorption of non-enzymatic substances on lignin. The molecular weight of these substances has a significant impact on their performance. Different strategies can be used for a good utilization of yeast cells in terms of biorefinery concept.

## Background

Expected shortage of fossil fuels and the concern for climate change has increased the interest for the production of fuels and chemicals from renewable sources. Ethanol is already being produced by the fermentation of sucrose (from sugarcane or sugar beets) or starch (from corn or wheat) carbohydrate-rich substances [1]. However, to meet increasing demands for fuel, and to avoid conflict between food and energy production, it will be necessary to use other kinds of biomass, and in this context, lignocellulosic materials have attracted interest as sources of fermentable sugars [2]. Furfural residue (FR) is an industrial waste in China. The corncobs are heated at high temperature ranged from 170°C to 185°C under acidic conditions to hydrolyze arabinoxylans into xylose, and then the xylose are converted into furfural. The cellulose and lignin in the cobs are relatively stable under these conditions, so the residues left over after the furfural production are enriched in cellulose (about 45%), which can be easily hydrolyzed by cellulases. Currently, FR is only recycled as boiler fuel, but a potential use could be as raw materials for biofuel production [3].

The utilization of lignocellulosic feedstocks for biofuel production requires the deconstruction of their polysaccharides to free sugars, mainly by acid and enzymatic hydrolysis. Enzymatic biodegradation is one of the most promising options for production of lignocellulosic biofuels as mild and environmentally friendly process. However at the same time, the lignin-carbohydrate complexes pose much challenge to decrease enzyme dosage in lignocellulose digestibility, while reducing the cost of ethanol production [4-6]. Other properties, including surface properties and crystallinity, impede enzymatic hydrolysis, which has been investigated in the literature recently [7].

It is well know that pretreatment is required to improve enzymatic digestibility on lignocellulose [2]. Previous studies have been showed that post-pretreatments and addition of some supplements increase the hydrophilicity of lignin [8-10]. Some examples are the hemicellulase supplementations which stimulate glucose release by depolymerisation of hemicelluloses [11,12]. Surfactants enhance release of free glucose by reducing the interaction between enzymes and lignin [13]. Non-protein surfactants contain synthetic surfactants as Tween-20, Tween-80, PEG 4000 and PEG 6000 [13-15], and natural surfactants as *Gleditsia* saponins [16]. Bovine serum albumin (BSA) is a protein surfactant and its enhancement is mainly due to surface activity than catalytic activities [11].

As the main microorganism for bioethanol production, yeast contains about 8% of nitrogen (equivalent to 50% of protein). Being rich in proteins attribute to that co-product of 1G-ethanol industry could be marketed as animal fodder and the feasibility of the use of spent cells as nutrient sources [17]. Occasionally, it was found that yeast hydrolysates (YH), obtained by hydrothermal treatment, can increase enzymatic digestibility of furfural residues (FR) [18]. In the current work, YH was used as a cheap supplementation of commercial cellulases and the components of YH were separated by centrifugation and size exclusion chromatography and then characterized. The aim of this study is to better understand hydrothermal treatment process of yeast, find out the mechanism for YH enhancement of enzymatic digestibility and evaluate the absorption capacity of YHL protein on lignin.

## Results

### Hydrothermal treatment of yeast

**Figure 1:**
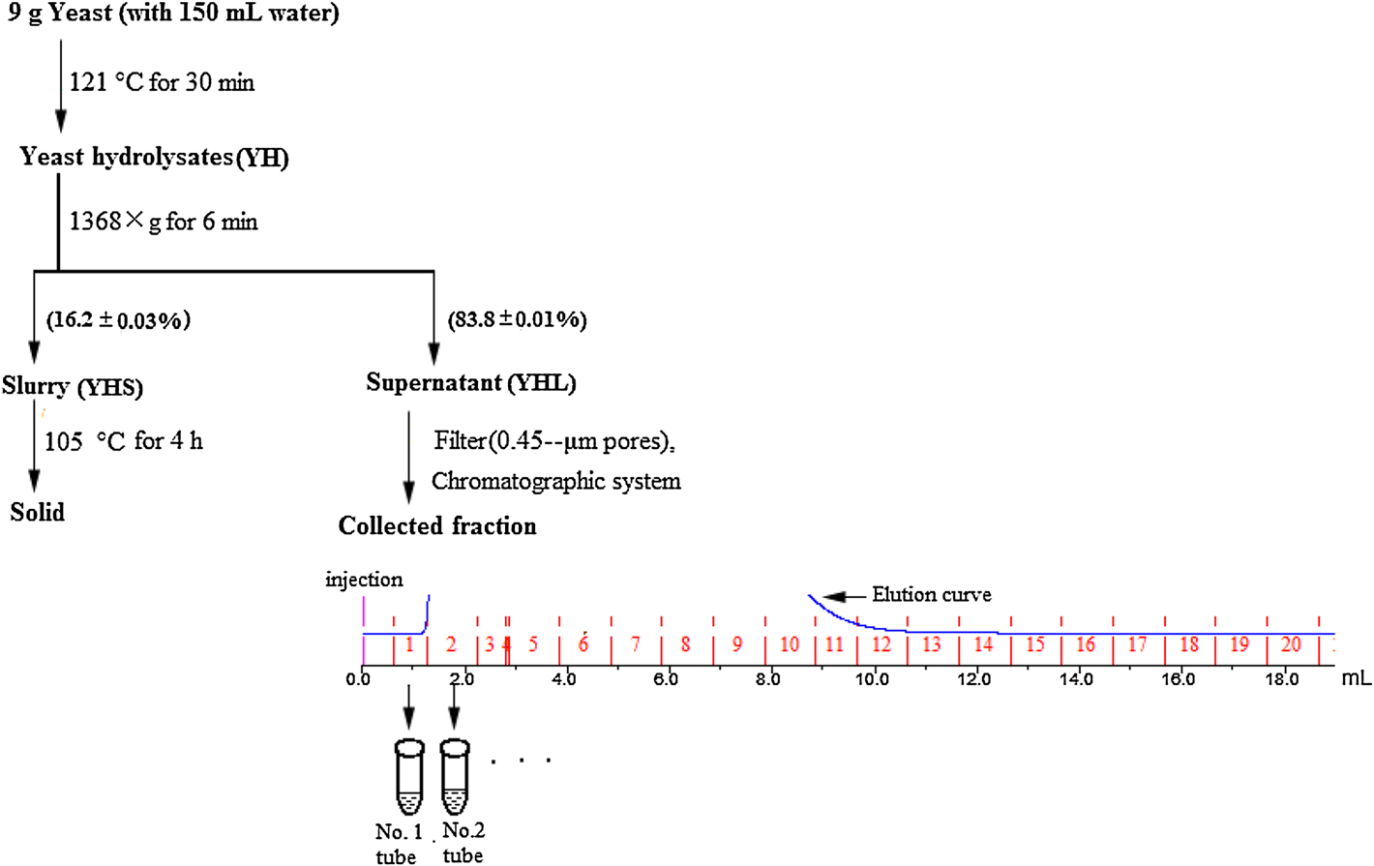
Scheme for separating components of yeast hydrolysate (YH).

After centrifugation, 159 g of YH was separated into two phases, 132.3 g of supernatant (YHL, 83.8%) and 24.6 g of slurry (YHS, 16.2%) (Figure 1). The total mass loss was about 1.4% after hydrothermal treatment and centrifugation. The dry matter content of YHS and dissolved matter content (DMC) of YHL were 23.5 and 2.07%, respectively. As seen in Figure 1, the nitrogen content of YHS was 7.25% and the protein concentration of YHL was 0.75 g/L, accounting for 2.28% of total yeast nitrogen. One explanation for the relative small nitrogen in YHL could be that there is nitrogen loss during hydrothermal treatment and some nitrogen may not be detected by the Bradford method. Soluble sugars were not detected in the YHL according to the HPLC analysis. The matter recovery after hydrothermal treatment and centrifugation was 94.6%.

### The effect of YHS on enzymatic hydrolysis and the relative roles of pH control and YHL addition

**Figure 2:**
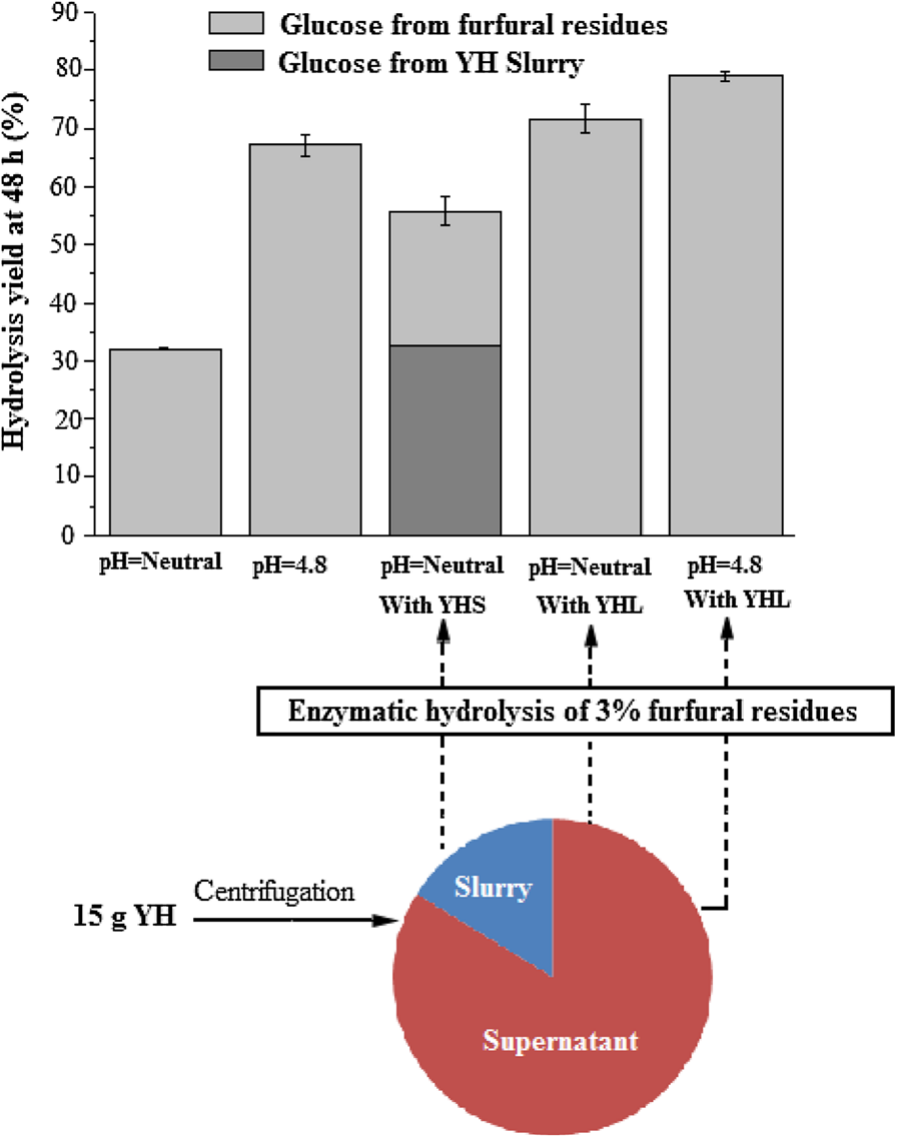
The effect of 2.43 g (YHS) and 12.6 g (YHL) on enzymatic hydrolysis of 3% furfural residues at 45°C.

For comparison, the addition of YHS and YHL in the FR was tested in hydrolysis experiments. YHL and YHS were separated by centrifugation, followed by being added to hydrolysis. The yield of enzymatic hydrolysis was calculated based on FR cellulose (Figure 2). Based on the datas of enzymatic hydrolysis of YH [18], when the YHS was supplemented for the FR hydrolysis, the glucose yield from FR was calculated to be 23.1%, which was lower than that without YHS and YHL (31.9%) at neutral pH (Figure 2). At neutral pH, the glucose yield with YHL (71.6%) was two times higher than that without YHS and YHL (31.9%) (Figure 2). Thus, these results could support that YHS had almost no effect on the digestibility [18].

The pH and YHL addition are likely to have an effect on the enzymatic hydrolysis of FR. In general, the optimized pH of enzymatic hydrolysis is well recognized to be 4.8. When neither of YHL and YHS was added, controlling pH at 4.8 increased the glucose yield from 31.9 to 67.2%. The addition of YHL shortened the gap between the glucose yields at different pH values (Figure 2). For instance, the yield of hydrolysis at neutral pH with YHL was higher than that at pH 4.8 without YHL. These results indicated that the interference of lignin on enzymes had much higher impact on enzymatic hydrolysis than non-optimum pH. The enzyme activity was not detected in YHL. When YHL was added to control experiments that use Cellulolytic Enzyme Lignin as sole substrate, Filter paper activity (FPU) in the liquid fraction samples at 4, 24 and 72 h were increased by 0.1%, 6.6% and 5.7%, respectively, indicating that YHL is a protein surfactant and its enhancement is mainly due to surface activity than catalytic activities [11,13].

### Comparison of molecular weight distribution between YHL and cellulases

**Figure 3:**
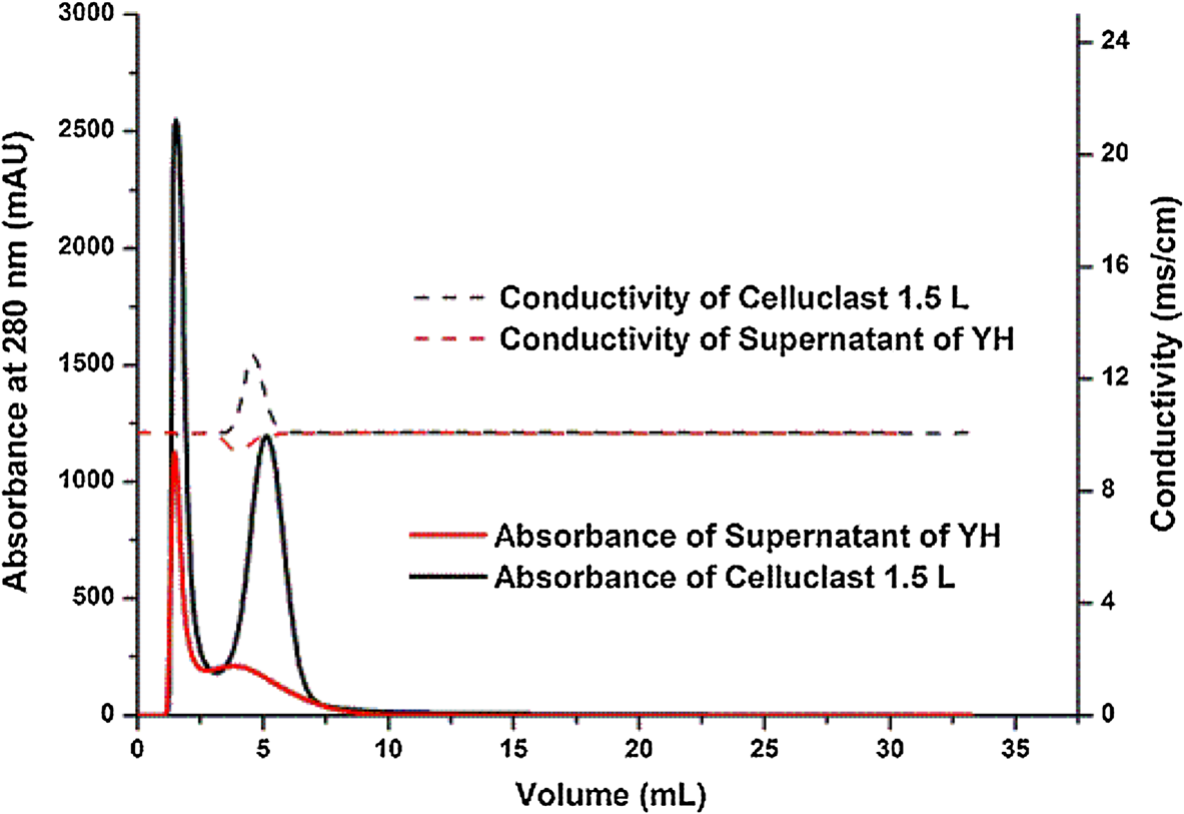
Comparison of the molecular weight distribution of cellulases and YHL.

As seen in Figure 3, two similar peaks were presented in elution curves (UV at 280 nm) of Celluclast 1.5 L and YHL, indicating that the components of cellulase and YHL showed a close molecular weight. It should be noted that the conductivity curve of Celluclast 1.5 L had a positive peak in retention time of 1.67 min (accumulated volume 5 mL) while an adverse peak was found for YHL. The elution curve of Novozym 188 was similar to that of Celluclast 1.5 L except for a straight line of conductivity curve (data not shown).

### Protein and mass distribution between fractions of YHL

**Figure 4:**
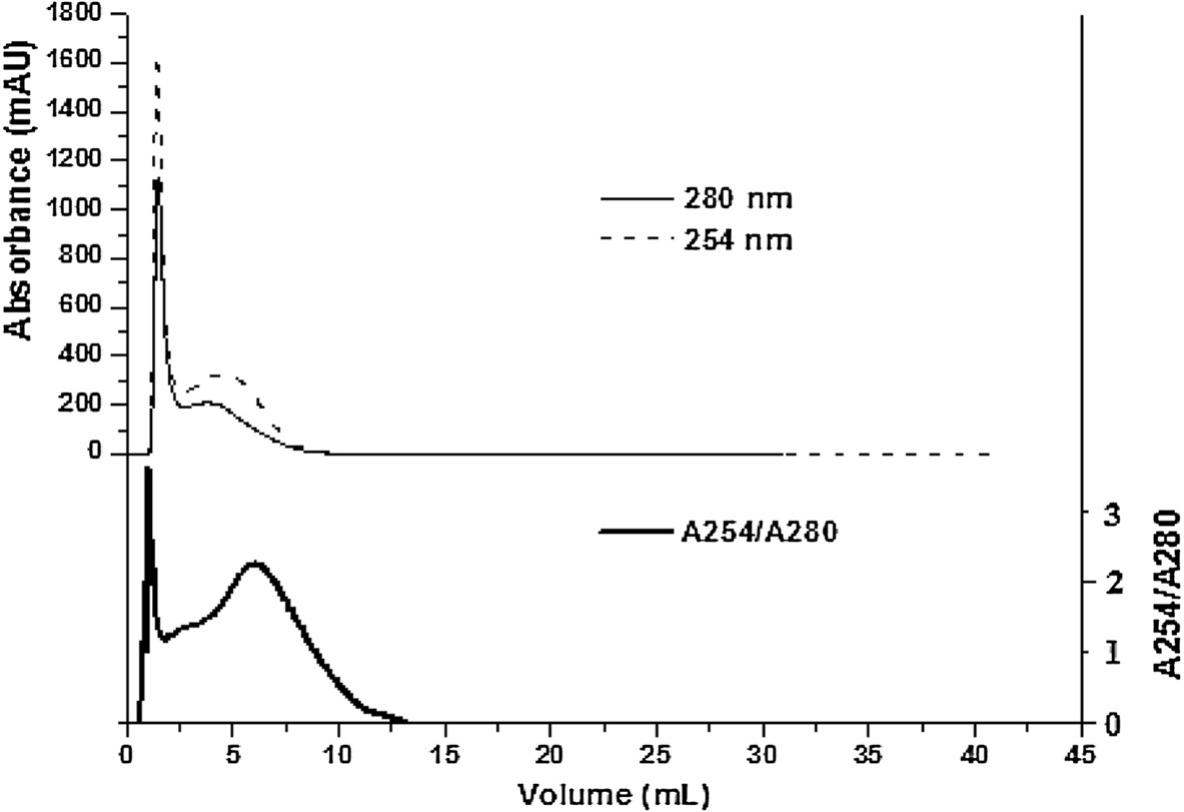
Comparison of elution curves of YHL at 254 and 280 nm.

**Figure 5:**
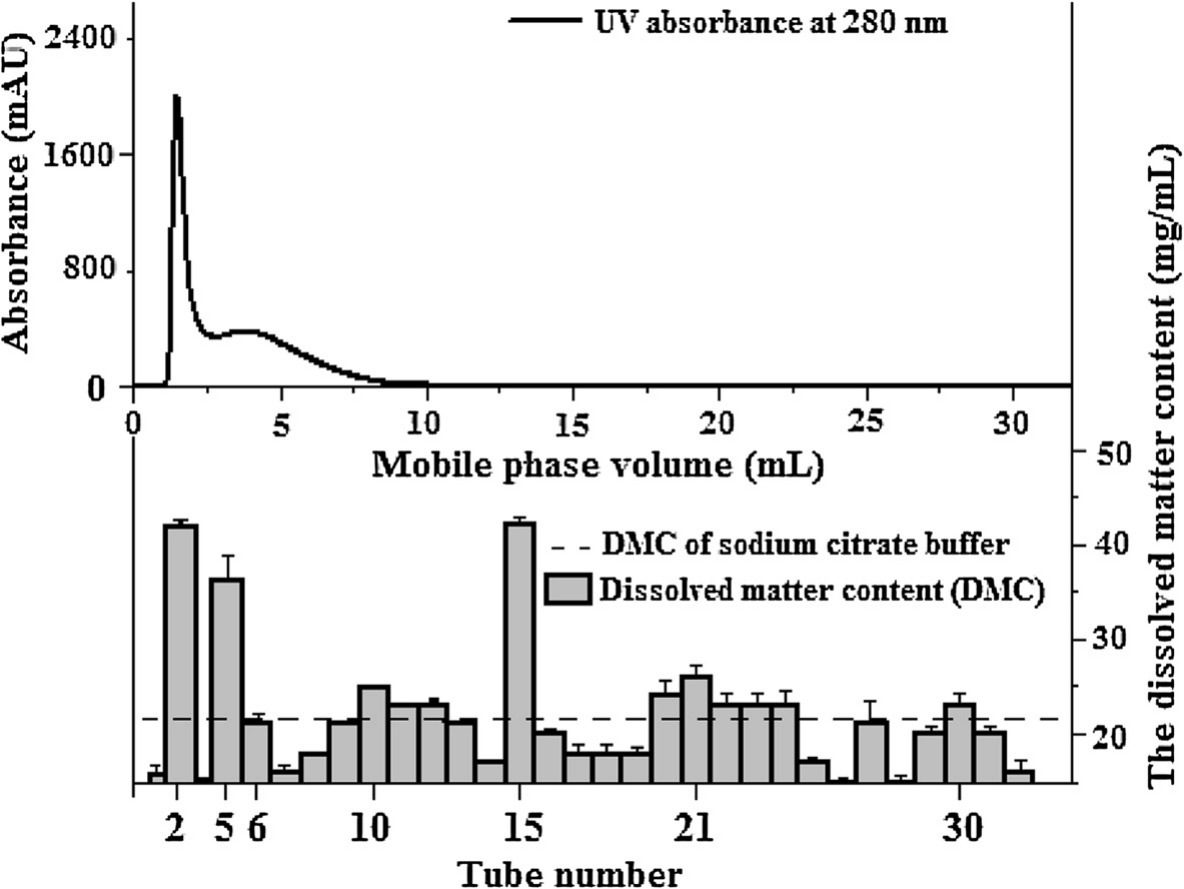
The total dissolved mater content (DMC) of fractions of YHL.

Fractions of YHL were collected in 32 tubes separately (Figure 1). The protein concentration of fraction in each tube was detected with the Bradford method, except a relatively small fraction, 0.15 mL, in tube 4 (Figure 1). Fraction in tube 2 contained 96.97 mg/L protein, accounting for about 51.8% of sample proteins. The protein concentration in tube 3 was out of linear range of the Bradford method and proteins were not detected in other tubes. Figure 4 shows the relative purity of protein in YHL at different UV elution curves (254 and 280 nm) [19,20]. The lowest A254/A280, about 1, was found at the top of peak 1 (elution volume of 1.48 mL), meaning that the purity of protein in tube 2 was the highest (Figure 4). These results were consistent with protein concentration by the Bradford method. A254/A280 increased with increasing mobile phase volume from 2.11 to 6.25 mL, indicating that the fractions in tube 5 and 6 contained certain amount of other UV-absorbing compounds (Figures 4 and 5). Those compounds are inferred to attribute to the adverse conductivity peak in Figure 3. However the molecular weight of proteins and other UV-absorbing compounds in tube 2, 5 and 6 showed the close molecular weight to components of Celluclast 1.5 L and Novozym 188.

DMC was detected to investigate the mass distribution between fractions of YHL as UV detector cannot determine substances without ultraviolet absorption (Figure 5). DMC of sodium citrate buffer is theoretically calculated to be 25.54 mg/mL. In practice, it was 21.68 mg/mL, showed as dotted line in Figure 5. One explanation for this could be that some reactions during the oven-dried process (105°C) decrease the value. Figure 5 shows that the DMC of fractions in tube 2, 5, 15, 21 and 30 were higher than 21.68 mg/mL. A close DMC was found between fractions in tubes 9, 13 and 27 and sodium citrate buffer, indicating that the minimum components of YHL were transferred into those tubes. Results demonstrated that substances with different molecular weight were formed during hydrothermal treatment and those of high molecular weight were predominating.

### The effect of YHL fractions with different molecular weight on enzymatic hydrolysis

**Figure 6:**
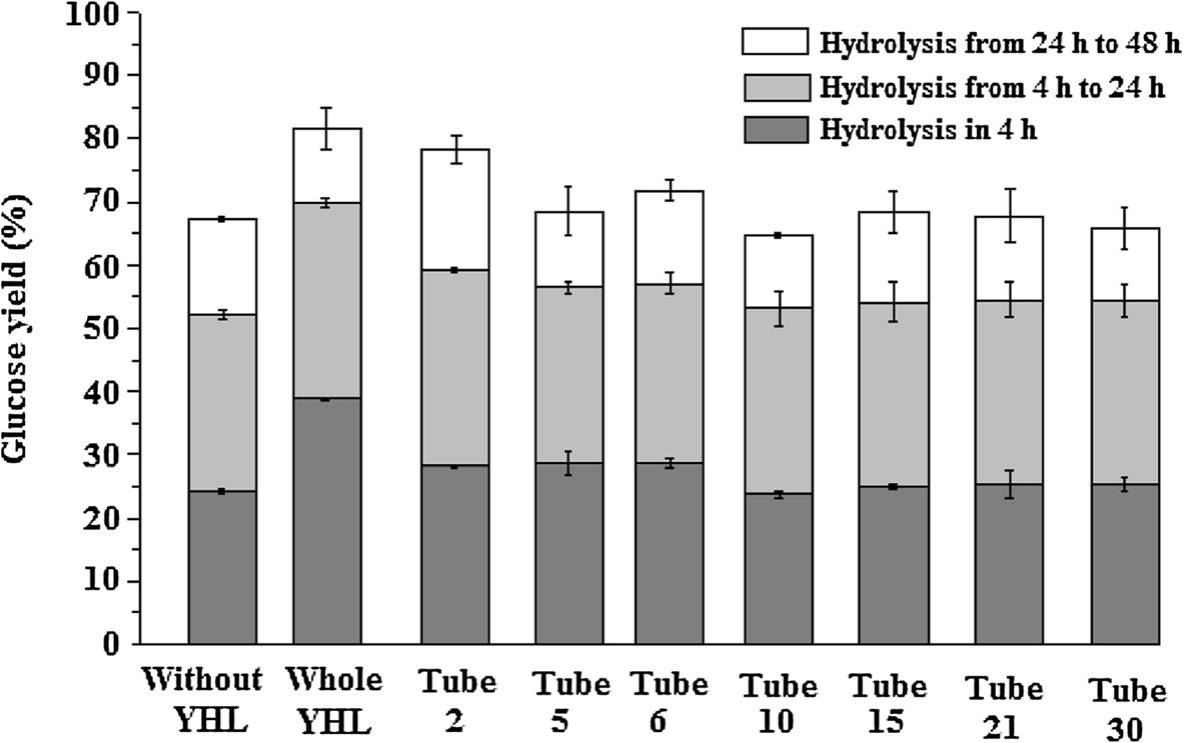
The effect of fractions of 6.29 g YHL on enzymatic hydrolysis of 3% of furfural residues at 45°C.

The effect of fractions in tubes 2, 5, 6, 10, 15, 21 and 30 on enzymatic hydrolysis were investigated at a relatively high DMC (Figure 5). As seen in Figure 6, the glucan digestibility was improved in tubes 2, 5 and 6 at 24 and 48 h. Fractions in tube 2 had a better performance than fractions between tubes 5 and 6. The highest concentration of protein is supposed to be the reason for the best performance of fraction in tube 2 [3]. After 72 h, the glucose yield fraction in tube 5 seems not be improved, probably due to that enzyme effectiveness dropped over time by heat-induced denaturation [21,22]. No significant effect of fractions eluting after 7.5 mL (tube 10, 15, 21 and 30) on hydrolysis was observed (Figure 6). The molecular weight of substances in fractions is supposed to have an effect on their performance. Hydrolysis with the whole YHL obtained the highest yield, indicating that there were a synergy between fractions in tube 2, 5 and 6. However when the amount of YHL was decreased from 12.6 g (Figure 2) to 6.29 g (Figure 6), the hydrolysis efficiency decreased slightly, which is consistent with the previous study [18].

### Competitive adsorption of non-enzymatic protein from YHL and cellulases on lignin

After hydrolysis of 3% FR, the nitrogen content of the dried residues was 0.84%. For that with YHL, it was 1.27%. There was 3.61 gram of liquid per gram of dried residue in the slurry. When YHL were used in enzymatic hydrolysis, the nitrogen in the residual liquid should be deducted to determine how many proteins were absorbed on lignin. Assuming that all nitrogen from yeast distributes in YHL and YHS and there is no nitrogen loss during hydrothermal treatment, the maximum nitrogen content of YHL is 2.05 mg/mL based on the total nitrogen of yeast and the amount of nitrogen of YHS. When YHL is diluted by 2 times in enzymatic hydrolysis (15 g YH were added to enzymatic hydrolysis with a total working weight of 30 g), the maximum nitrogen content in liquid phase is 1.03 mg/g hydrolysate. A 3.61 gram of hydrolysate contained 3.72 mg of nitrogen. The amount of nitrogen absorbed on lignin was indirectly estimated by subtracting the amount of free nitrogen in the residual hydrolysate. More than 0.63 mg nitrogen from YHL (12.7-8.35-3.72 = 0.63 mg) was absorbed on 1 gram of lignin. Competitive adsorption of non-enzymatic proteins and cellulases on lignin is probably a good reason for the enhancement of glucose yield [4,13].

## Discussion

The hemicellulase addition is not suitable for hydrolysis of materials that contain a little hemicellulose content as FR. The supplementations of commercial cellulases with other enzymes as hemicellulases that contribute novel catalytic activities are simple methods to enhance the fermentable sugar yields. Lignin controlled cellulose accessibility by restricting xylan accessibility to some extent, so hemicellulases promote enzymatic hydrolysis of cellulose by depolymerisation of hemicelluloses [11]. However, the pretreatment severity, fermentation mode and substrate composition would affect the efficiency of hemicellulase addition [12]. The xylosidase enhancement was not a general protein effect because BSA at similar concentration did not increase hydrolysis yields from corn stover [11]. The xylanases supplementation was observed to be only positive effect in SHF (separate hydrolysis and fermentation) but not in SSF (Simultaneous saccharification and fermentation) mode [5].

Lignin poses a bigger challenge to low enzyme dosage during the enzymatic hydrolysis process than hemicelluloses. Two strategies, including increasing the hydrophilicity of lignin and removing lignin by post-treatment, have been employed in reducing the interference of lignin on enzymes [8,15]. When different substrates are used, quantifying the relative roles that lignin might play in either binding the enzyme or limiting the swelling of the cellulose is helpful to decide which methods should be used [4]. Besides the end product type affects the severity of post-treatment and the degree of delignification [23]. The surface active additives are the most common supplementations used during hydrolysis and pretreatment process [15]. According to a previous study, addition of 2.5-5.0 mg/mL (123–245 mg/g of the substrate) of BSA was found to be a highest digestibility in pretreated softwood [4]. Other study showed an optimum BSA concentration of 25 mg/g DM using wheat straw [13]. Both studies suggested that BSA to reduce the adsorption of cellulases as surfactant on enzymatic hydrolysis. For instance, the maximum adsorption capacities of pretreated corn stover were ranged from 38.7 to 67.5 mg/g cellulose (corresponding to 77.8-124.6 mg/g substrates) [21]. In this study, the protein concentration used in this study ranged from 0 to11.77 mg/g FR. Since non-protein substances (fractions in tube 5 and 6) could enhance the sugar yield simultaneously, it is inferred that the potential sites in FR were not occupied by proteins completely, meaning that the optimum protein range of FR would be higher than 11.77 mg/g FR. However, although these sites were not all associated with proteins, further additions increased hydrolysis yield slightly, indicating that non-protein substances played an important role in enhancement of sugar yield. Interestingly, it has been found that the levelling off concentration of the surface active performance of YHL was about 30 g/L (15 g YH were added to enzymatic hydrolysis with a working weight of 30 g) [18]. Possibly, it is difficult to improve the performance of YHL further only by changing the severity of hydrothermal treatment. The use of diluted acid can improve the performance of YH as nutrients but may decrease the molecular weight of product, thus resulting in losing the function of enhancement of enzymatic hydrolysis [17].

This study showed that fermentation residues from bioethanol process can be reused to produce YHL or proteins that can improve enzymatic hydrolysis. The process had such advantages that there is no need to dry YHL and to deal with storage and transport, thus resulting in less production cost. When byproduct of dry-mill starch to ethanol production is used, the majority of yeast nitrogen will be dissolved in YHS, thus indicating that the feasibility of marketing YHS as animal fodder. Other studies gave methods to utilize carbohydrates in YHS [3-27]. Integration process of 2G bioethanol with 1G has been proved to be competitive with 1G bioethanol [25]. This study could provide a potential way to utilize the fermentation residues, which is difficult to be marketed as animal fodder [3].

The YHL supplementation is supposed to be positive effect on SSF mode. Hydrolysis experiments revealed that there were few inhibitors produced during hydrothermal treatment. The addition of YHL will reduce the decrease of enzyme activity due to non-optimal pH in SSF as the interference of lignin on enzymes has much higher impact on enzymatic hydrolysis than the decrease of enzyme activity due to non-optimum pH. Besides YHL are inferred to be nutrients for fermentation according to a previous study [18]. Further investigations are needed, however, to use SDS-PAGE and mass spectrometry for characterization of the elution fractions to balance the relative contribution of YHL as fermentation nutrients and hydrolysis promoter. Besides more works are needed to investigate the effect of substances with low molecule weight on the impurities of fermentation broth and product purification cost, especially for lactic acid fermentation [28].

## Conclusions

The majority of nitrogen and carbohydrate of yeast cells were transferred into YHS during hydrothermal treatment. The increase in enzymatic convertibility of FR is only due to YHL. There were soluble substances of different molecule weight in YHL and those of high molecular weight were predominating, including non-enzymatic proteins and other UV-absorbing compounds, which showed close molecule weight to components of cellulases. Supplementation of cellulases with YH enhanced enzymatic convertibility mainly by competitive adsorption of proteins and cellulase on lignin. Since YH can be used as nutrients for fermentation, different strategies can be used for a good utilization of yeast cells in terms of biorefinery concept.

## Methods

### Raw material preparation

Raw furfural residue was kindly provided by Chunlei Company (Hebei province, China). The initial pH of the material was between 2 and 3. After raw furfural residue was water-rinsed until neutral pH and then dried at 60°C for 12 h. *Saccharomyces cerevisiae* was used in the form of dry yeast (Angel Yeast Company Ltd, China).

### Fractionation of yeast hydrolysate (YH)

Nine grams of yeast cells were added into a 180 mL-sealed pressure flask. Each flask was contained 150 mL of distilled water and then autoclaved at 121°C for 30 min [18]. The YHS and YHL were collected by centrifugation at 1368 × g for 6 min. The determination of moisture content of YHS was made in an oven-dried at 105°C (Figure 1). The pH of YHL was about 6. A 1 mL supernatant was oven-dried (105°C) and the residual solid was quantified to determine DMC of aqueous phase. DMC were calculated as follows:

$$\mathrm{DMC}\;\left(\mathrm{mg}/\mathrm{mL}\right)=\frac{\text{The}\;\text{residual}\;\text{solid}\;\text{after}\;\text{oven}‒\text{dried}\;\left(\mathrm{mg}\right)}{\text{The}\;\text{liquid}\;\text{volume}\;\left(\mathrm{mL}\right)}\times 100\%$$

### Component separation of YHL

Before separation, YHL was filtered using 0.45-μm filters. The separation of each component was made on an AKTA purifier (Amersham Pharmacia Biotech; Uppsala, Sweden) equipped with a pump (P-920), UV monitor (UPC-900), a valve (INV-907), a mixer (M-925), and a fraction collector (Frac-920). The separation was performed in a 5 mL HiTrap desalting size exclusion column at room temperature with 3 mL/min of sodium citrate buffer (pH 4.80 and 50 mM) as mobile phase. The injection volume was 0.5 mL and injections were carried out 14 times to separate 6.29 g of YHL (7.5 g YH contains 6.29 g YHL). When there is no peak, Frac-920 collects 1 mL fraction in each tube (Figure 1). When there is a peak, the peak will be collected as one large fraction or in several tubes each collecting at most 1 mL fraction (Figure 1). A 0.33 mL of Celluclast 1.5 L was added to a 10-mL volumetric flask and diluted with sodium citrate buffer to obtain 40 g/L solution for component separation.

### Enzymatic hydrolysis

The enzymatic hydrolysis (EH) of furfural residue was performed using Celluclast 1.5 L (with activity of 75 FPU/mL) and supplemented with the β-glucosidase preparation Novozym 188 (43.9 IU/mL). Both enzyme preparations were provided by Novozymes A/S, Bagsvaerd, Denmark. The filter paper units (FPU) were determined according to the IUPAC method [29] and β-glucosidase activity by [30]. The EH was made using 3% FR in 60-mL conical flasks with a working weight of 30 g. The amount of Celluclast 1.5 L and Novozym 188 added corresponded to a 12.5 FPU and 13.5 IU/g cellulose of FR, respectively. The flasks were kept in a shaker bath at a temperature of 45 ± 0.1°C at 150 rpm. YHL was used in hydrolysis experiments at one of two scales. Small and large scale hydrolyses were done using 6.29 and 12.6 g of YHL, respectively. The initial reaction mass was kept 30 g by adding sodium citrate buffer (pH 4.80 and 50 mM). After 144 h, the insoluble phase of hydrolysate (mainly rich in lignin) was collected by centrifugation at 1368 × g for 6 min and then oven-dried at 105°C. Duplicate samples were withdrawn at 0, 4, 6, 24 and 48 h for carbohydrate analysis by HPLC. Liquid fractions were separated by filtration and subsequently stored at −18°C awaiting analysis.

In control experiments, EH were carried out by using 0.425 gram of cellulolytic enzyme lignin as sole substrate with or without 6.29 g of YHL. Duplicate samples were withdrawn at 4, 24 and 72 h for FPU analysis.

### Cellulolytic enzyme lignin isolation

Enzyme hydrolysis of FR was carried out as above without the addition of YHL. The hydrolysate at 96 h was filtered and the residues were collected for a second hydrolysis. After the second hydrolysis using identical amounts of cellulase and β-glucosidase, the residues were recovered by filtration and washed with 400 mL of distilled water (about 50°C), followed by aqueous dioxane extraction of ball-milled wood meal [31]. The final lignin was put into vacuum oven and dried at room temperature. The lignin samples were ground and screened through 40 mesh.

### Compositional analysis

The chemical composition of water-rinsed furfural residue (FR) and yeast were analysed and prepared according to the standard procedure by NREL [32]. FR composition on an original dry weight (DW) residue basis was glucan, 48.2%; Klason lignin, 43.3%; and ash, 6.42%. Yeast has a composition of glucan, 18.4%; protein, 48.1%; and mannan, 2.77%. The total nitrogen content of YHS and the lignin residue was determined by the conventional Kjeldahl method [33]. The protein content was obtained by multiplying the elemental N content by the universal factor of 6.25. The protein content of YHL was measured with the Bradford method using BSA as a standard [34].

### Sugar analysis

Previous analysis, the hydrolysates were centrifuged at 1368 × g for 6 min and filtered using 0.20-μm filters. Monosaccharides were quantitatively determined on an Aminex HPX-87P column at 85°C with 0.6 mL/min water as eluent. The HPLC system used as a Waters 2695e (Milford, MA, USA), comprising a Waters performancePLUS vacuum degasser, a Waters four channels solvent delivery system, a Waters high temperature column oven, a Waters 2414 refractive index (RI) detector and a Waters plus auto-injector. All experiments were performed in duplicates, and the analysis was carried out at least three times for each sample.

## Abbreviations

YH: Yeast hydrolysates; FR: Water-rinsed furfural residue; YHS: The solid phase of yeast hydrolysates obtained by centrifugation; BSA: Bovine serum albumin; YHL: The liquid phase of yeast hydrolysates obtained by centrifugation; DMC: Dissolved matter content; SSF: Simultaneous saccharification and fermentation; SHF: Separate hydrolysis and fermentation; EH: Enzymatic hydrolysis; FPU: Filter paper activity.

## Competing interests

The authors declare that they have no competing interests.

## Authors’ contributions

YT, ZL and FL designed and coordinated the study. YT carried out all the experiments. YT and HY analysed the results. YT wrote the paper, and JJ, FL and CC were reviewed the manuscript. All authors read and approved the final manuscript.
